# Glomerular diseases and cancer: evaluation of underlying malignancy

**DOI:** 10.1007/s40620-015-0234-9

**Published:** 2015-10-26

**Authors:** Antonello Pani, Camillo Porta, Laura Cosmai, Patrizia Melis, Matteo Floris, Doloretta Piras, Maurizio Gallieni, Mitchell Rosner, Claudio Ponticelli

**Affiliations:** 1 Department of Nephrology and Dialysis, G. Brotzu Hospital, Piazzale Ricchi, 1, 09134 Cagliari, Italy; 2 Medical Oncology, I.R.C.C.S. San Matteo University Hospital Foundation, Pavia, Italy; 3 Nephrology and Dialysis Unit, Istituti Ospedalieri Cremona, Cremona, Italy; 4 Nephrology and Dialysis Unit, Ospedale San Carlo Borromeo, Milan, Italy; 5 Division of Nephrology, University of Virginia Health System, Charlottesville, VA USA; 6 Nephrology and Dialysis Unit, Humanitas Clinical and Research Center, Rozzano, Milan, Italy

**Keywords:** Glomerular diseases, Cancer, Onconephrology, Nephrotic syndrome, Immunosuppressive therapy, Membranous nephropathy

## Abstract

Onconephrology is an emerging medical subspecialty focused on the numerous interconnections between cancer and kidney diseases. Patient with malignancies commonly experience kidney problems including acute kidney injury, tumor lysis syndrome, fluid and electrolyte disorders and chronic kidney disease, often as a consequence of the anti-cancer treatment. Conversely, a number of glomerulopathies, tubulopathies and vascular renal diseases can early signal the presence of an underlying cancer. Furthermore, the administration of immunosuppressive drugs, especially cytotoxic drugs and calcineurin inhibitors, may strongly impair the immune response increasing the risk of cancer. The objective of this review article is to: (i) discuss paraneoplastic glomerular disease, (ii) review cancer as an adverse effect of immunosuppressive agents used to treat glomerulopathies, and (iii) in the absence of international approved guidelines, propose a screening program based on expert opinion aimed at guiding nephrologists to early detect malignancies during their clinical practice.

## Introduction

Onconephrology is a new discipline that covers the many interrelations between cancer and kidney diseases [1]. Acute kidney injury, tumor lysis syndrome, fluid and electrolyte disorders and chronic kidney disease are frequent complications of anti-cancer treatment, particularly in elderly patients [2]. On the other hand, a number of glomerular, tubulo-interstitial and vascular renal diseases can be associated with solid or hematopoietic malignancy [3] and may often represent the first clinical manifestation of an underlying cancer. This is of particular concern for the nephrologist, not only because it can lead to delayed diagnosis of cancer but also because incorrect diagnosis may lead to harmful treatment. Lastly, an already existing occult cancer that is recognized too late may be wrongly attributed to the immunosuppressive therapy used to treat the original, presenting renal disease.

In this article, we will discuss the most frequent glomerular diseases that are caused by cancer (paraneoplastic glomerulopathies), the oncogenic role of immunosuppressive therapy, and screening recommendations for detecting cancer in patients with glomerulopathies.

## Paraneoplastic glomerulopathies

The term paraneoplastic syndrome was introduced to indicate the clinical manifestations that are not directly related to tumor burden, invasion, or metastasis but are caused by the secretion of tumor cell products, such as hormones, growth factors, cytokines and tumor antigens [4]. There is experimental evidence showing that tumor-bearing animals can develop proteinuria and glomerular lesions with abundant immunoglobulin (Ig)G deposits in the glomeruli and effacement of podocyte foot processes [5]. In 1922, Galloway introduced the concept of paraneoplastic glomerulopathy [6], but the first original study highlighting the association between cancer and nephrotic syndrome (NS) was published in 1966 by Lee and colleagues [7].

It is difficult to assess the true prevalence of cancer-related glomerulopathies due to a number of confounding factors such as: (i) potential detection bias (e.g., patients with membranous nephropathy are likely to be more aggressively screened for cancer); (ii) demographic characteristics of the population (e.g., both membranous nephropathy and cancer occur more frequently in the elderly and/or in heavy smokers); and (iii) most of the agents used to treat glomerular disease are potentially oncogenic drugs, which may themselves lead to subsequent malignancies [4]. Thus, it is not surprising that little information about the prevalence of paraneoplastic glomerulopathies is available.

The prevalence of paraneoplastic glomerulopathies has been evaluated by a few retrospective, and a few prospective, studies. Pai and colleagues [8] selected 120 patients with different types of primary glomerulonephritis (GN) and found that 17 (14.1 %) had cancer. Among them, six were diagnosed at the time of biopsy, four in the first year, and seven after 1 year. The histological renal diagnoses included membranous nephropathy (MN), membranoproliferative GN (MPGN), crescentic GN, and focal segmental glomerulosclerosis (FSGS). Cancer was detected by screening that included a chest x-ray and abdominal ultrasound [8]. In the Tromso study, initially aimed at combating the high mortality of cardiovascular disease in Norway and then extended to investigate the causes and possible prevention of other chronic diseases, Jorgensen and colleagues examined the possible relationship between cancer and albuminuria in a prospective cohort of 5425 participants with no history of diabetes, cancer or macroalbuminuria. The participants with an albumin-to-creatinine ratio in the highest quintile were 8.3 and 5.4 times more likely to develop bladder and lung cancer, respectively, than those in the lowest quintile [9]. Saitoh and colleagues analyzed the risk of GN in 125 patients with myelodysplastic syndrome. Five patients (4 %) had glomerular disease and three (2.4 %) had nephrotic syndrome [10]. Birkeland and colleagues published a retrospective study that analyzed 1958 patients found by matching the Danish renal biopsy registry with the oncological registry [11]. None of the participants with renal disease had cancer at the time of biopsy. These patients had histological renal diagnoses of different types of primary GN with extra or endocapillary proliferation and sclerosis. During follow-up, 102 patients developed cancer (5.2 %). Cancer localization in males included lung, skin, lymphatic and hematopoietic tissue, with diagnoses of MN, MPGN, and proliferative endocapillary GN at renal biopsy. In women, cancer localization included the gastrointestinal tract and lymphatic and hematopoietic disorders with a prevalence of minimal change disease (MCD) at renal biopsy. The risk of cancer among patients with biopsy-proven GN was 2.5 and 3.5 times higher than in the general population at 1 and 2 years, respectively. However, at 5 years or more after biopsy the risk of cancer was the same [11]. Rihova and colleagues selected 129 patients with a histological diagnosis of MN and found 8 patients with tumors (6.2 %). Five were identified at the time of biopsy, which was accompanied by chest x-ray, abdominal ultrasound scan, serum tumor markers and mammography in patients >50 years of age. The most commonly found tumors were affecting lung, colon, and prostate [12]. Lefaucheur and colleagues selected 240 patients with a histological diagnosis of MN. Among them, 24 patients (10 %) had cancer, which was more often localized in the lung, prostate and stomach [13]. Twenty-one cases of cancer were detected at the time of renal biopsy, the remaining three within 1 year post-biopsy. There were no differences between men and women, but a higher incidence was reported in the elderly and in heavy smokers [13] (Table 1).

**Table 1:** Literature reporting evidence of cancer-related glomerulopathies

Authors	No. patients	Cancer (%)	GN	Type of cancer	Time of diagnosis	Screening
Birkeland [11]	1958	102 (5.2)	MN, MCD, MPGN, diffuse endocapillary GN	Colon, lung, skin, lymphatic and hematopoietic tissue	<1 year from biopsy: 27 1–4 years from biopsy: 53 >4 years from biopsy: 22	ND
Zeng [49]	390	12 (3.1)	MN	LCDD, thyroid, GI, mediastinal	ND	Serum tumor markers
Lefaucher [13]	240	24 (10.0)	MN	Lung, stomach, prostate	At the time of biopsy: 21 during follow-up (max 1 year): 3	ND
Ehrenreich [48]	167	3 (1.8)	MN	ND	ND	ND
Bjorneklett [38]	161	33 (20.5)	MN	Lung, colon-rectum, prostate	Nine cases before diagnosis of MN three cases <6 months after biopsy	ND
Abe [47]	137	2 (1.5)	MN	ND	ND	ND
Rihova [12]	129	8 (6.2)	MN	Lung, colon, prostate	Five at the time of biopsy	Chest x-ray, abdominal ultrasound, mammography for age >50 years, serum tumor markers
Pai [8]	120	17 (14.1)	MN, MPGN, crescentic GN, FSGS	Bronchogenic, GI, breast	Six at the time of biopsy, four within 1 year	Chest X-ray, abdominal ultrasound
Cahen [50]	82	4 (3.2)	MN	ND	ND	ND

*MN* membranous nephropathy, *MCD* minimal change disease, *MPGN* membrano-proliferative glomerulonephritis, *LCDD* light chain deposition disease, *FSGS* focal segmental glomerulosclerosis, *GI* gastrointestinal, *ND* not detected

An association between cancer and glomerular disease is possible and it is probably related to altered immune responses in the presence of a malignancy [14]. Studies on murine models documented that T-helper 2 polarization has an important role in the development of thymoma-associated glomerular lesions in MCD and FSGS and an overexpression of interleukin (IL)-13, a T-helper 2 cytokine, induces MCD in rats [15, 16]. Furthermore, it is known that tumoral antigens can induce anti-tumor antibodies and consequently immune complex deposition in the glomeruli (sub-epithelial deposition in MN) [17, 18]. However, the diagnosis of paraneoplastic glomerulopathy is problematic due to the possible biases listed above and to the difficulty in identifying the tumor when GN is diagnosed (delayed diagnosis of malignancy). The sequence of events in the patient’s clinical history can help in differentiating a paraneoplastic glomerulopathy from malignancy caused by treatment of the GN. After cancer is diagnosed, a careful retrospective investigation of the radiological findings can also help in detecting small lesions that could have been misinterpreted. It is important to establish whether GN occurred in the presence of malignancy since ablation of cancer may result in remission of glomerular lesions.

Although MN is the most frequent GN associated with solid tumors, and MCD is the most frequent glomerular disease associated with Hodgkin lymphoma, many exceptions exist [19]. In fact, other forms of glomerular diseases, including FSGS, MPGN, IgA nephropathy (IgAN) and rapidly progressive GN may also be associated with solid tumors. On the other hand, not only MCD, but also MN, MPGN, FSGS and IgAN may be associated with hematologic malignancies [14]. Thus, specific tumors are not necessarily associated with a specific type of GN.

## Oncogenic role of immunosuppressive therapy

Any form of treatment that reduces immune surveillance may increase the risk of cancer. However, the role of single immunosuppressive drugs in increasing cancer risk is still under debate.

Glucocorticoids are not classified as oncogenic drugs [20]. However, these agents blunt the capacity of the immune system to mount a response by interfering with inflammation [21], inhibiting antigen presentation, suppressing cell-mediated immunity and partially inhibiting humoral immunity [22]. Thus, it is likely that the duration and dosage of glucocorticoid treatment would lead to a state of immunodeficiency, which might facilitate a faster development of a pre-existing neoplasia.

Alkylating agents are derived from nitrogen mustards. In clinical nephrology, the most frequently used alkylating agents are cyclophosphamide and chlorambucil. They share the ability to contribute alkyl groups to biologically active macromolecules such as DNA. Both cyclophosphamide and chlorambucil are classified as carcinogenic drugs. Two types of cancer are especially frequent with cyclophosphamide administration: bladder cancer and hematological malignancies. The development of bladder cancer is likely related to the chronic mucosal inflammation and irritation caused by acrolein, an inactive metabolite of cyclophosphamide, and/or to a direct oncogenic effect of cyclophosphamide or its metabolites on the urothelium. The risk of bladder cancer seems to be related to the cumulative dose of cyclophosphamide. In a Danish report, the standardized incidence ratio was found to be 9.6 times greater for patients who received > 36 g of cyclophosphamide as compared to those who received lower doses or no cyclophosphamide at all [23]. To prevent the possible development of bladder carcinoma, abundant fluid intake and sodium 2-mercaptoethanesulphonate (MESNA) should be prescribed concomitantly. MESNA binds to acrolein and prevents direct contact with the urothelium. It is currently recommended together with hydration in patients who receive intravenous high-dose cyclophosphamide [24]. However, some patients may develop hemorrhagic cystitis despite treatment with MESNA, because cyclophosphamide may cause two waves of apoptosis, one being independent of MESNA. In these patients, the anti-oxidant acrolein may be added to MESNA to reinforce a protective effect [25]. An increased risk of hematological malignancies with the prolonged use of cyclophosphamide has been reported; in patients with systemic lupus erythematosus, the risk of lymphoma was found to be higher with exposure to cyclophosphamide and high cumulative doses of steroids [26]. In granulomatosis with polyangiitis, a >36 g dose of cyclophosphamide resulted in a standardized incidence ratio of 59 for acute myeloid leukemia as compared to the general population [23]. As a prudent recommendation when using cyclophosphamide in a patient with GN, the cumulative dose should not exceed 360 mg/kg, i.e., 2 mg/kg/day for 6 months or 1 mg/kg/day for 12 months.

A marked increase in acute leukemia has been observed with long-term chlorambucil treatment in patients with polycythemia vera [27] or ovarian cancer [28]. Little information about the oncogenic effect of chlorambucil in renal patients is available. In a study of meta-analysis on 1504 children with idiopathic NS who received cyclophosphamide or chlorambucil, 14 (0.9 %) cases of malignancy were reported. In those patients, high doses of either one of the two alkylating agents were used. This meta-analysis also showed that chlorambucil has higher rates of severe side effects and should be considered a second-line drug [29]. A review of the data from three randomized trials in adults with idiopathic MN showed that the risk of cancer for patients who were treated with chlorambucil for ≤3 months was similar to that for the general population [30]. Although there is no clear cut-off for the doses of chlorambucil, we recommend not exceeding daily doses of 0.1–0.2 mg/kg and not prolonging treatment for more than 3 months.

Azathioprine is a thiopurine derived from 6-mercaptopurine. Azathioprine is a prodrug that is metabolized to thioinosinic acid and 6-thioguanine. These metabolites interfere with the de novo, and salvage, pathways of purine synthesis, respectively. The notion that long-term treatment with azathioprine can favor the development of neoplasia in organ transplant recipients has been known for many years [31, 32]. On the other hand, little information is available about the oncogenic effects of azathioprine in autoimmune diseases. A systematic review on the use of azathioprine in multiple sclerosis suggested that the risk of cancer was related to treatment duration of 10 years and a cumulative dose above 600 g [33]. The risk of cancer with azathioprine in inflammatory bowel disease has been differently estimated. Data from a Danish registry reported that azathioprine use was associated with increased risk of lymphoid tissue cancer and urinary tract cancer, the rate ratios being 2.40 and 2.84, respectively [34]. Sporadic cases of cancer in patients given azathioprine because of glomerulonephritis have been reported, but no systematic reviews are currently available.

Mycophenolic acid (MPA) inhibits the enzyme inosine monophosphate dehydrogenase by which guanine is synthesized from inosine. As a consequence the de novo pathway of purine synthesis is inhibited. Unlike azathioprine MPA is not incorporated into DNA. Since activated lymphocytes rely more than other cells on de novo pathways, T and B cells are preferentially affected by MPA, which causes an accumulation of lymphocytes at the G1–S phase of the cell cycle. There are two salts of MPA, mycophenolate mofetil (MMF) and an enteric-coated formulation of sodium mycophenolate. Although MPA may theoretically favor an increased risk of tumor through impairment in the immune surveillance, it has been shown to have antiproliferative activity against leukemia and lymphoma and an anti-tumor effect against colon and prostate cancer. In renal transplant recipients, the use of mycophenolate is associated with a reduced incidence of lymphoproliferative disorders [33, 35, 36].

Calcineurin inhibitors (CNI) are drugs that can inhibit the activation of T cells by interfering with the synthesis of IL-2 and other cytokines. Both cyclosporine and tacrolimus can increase the risk of cancer in transplant recipients [32]. In comparison with azathioprine, patients treated with CNI seem to develop neoplasias earlier and to be more susceptible to lymphoproliferative disorders and Kaposi sarcoma [37]. The risk of cancer after exposure to CNI depends on the dosage and duration of treatment. There are no data concerning the prevalence of cancer in CNI-treated patients with GN. It is possible that the risk of cancer may be relatively low, since administration of either cyclosporine or tacrolimus rarely exceeds 2 years in patients with glomerular diseases. When treatment is longer the administered doses are usually low.

In summary, any type of therapy weakening the immune response may theoretically render a patient more susceptible to cancer. The risk is higher with the use of carcinogenic drugs, and is also related to the intensity of immunosuppression and duration of immunosuppressive treatment. However, differential diagnosis between a paraneoplastic glomerulopathy and cancer caused by GN treatment can be difficult, mainly because GN can precede the identification of an underlying malignancy by months or even years. In either case, surveillance and a high degree of clinical suspicion for underlying cancer should be maintained in the patient with GN.

## Oncological screening for paraneoplastic glomerulopathies

How and whether patients with an established diagnosis of glomerular disease should be screened for cancer is still a matter of discussion among nephrologists. Currently, no guidelines have been recommended by renal scientific societies. We feel that a focused work-up would allow us to identify the majority of cancers in these patients. It should include a complete family and patient history, a careful physical examination including testicular or breast examination, and some investigations that are routinely prescribed in hospitalized patients such as: complete blood count, prothrombin (PT), partial thromboplastin time (PTT), electrolytes, uric acid, and renal and liver function tests, baseline viral titers, chest x-ray, renal and urinary tract ultrasonography, careful testing of urine sediment and a search for fecal occult blood. In the absence of a specific suspicion of cancer, oncological screening is usually the same as it is for the general population (except for chest computed tomography [CT] in heavy smokers). The work-up could be more extensive if the patient is older than 60 years of age, is a heavy smoker, and/or has nephrotic syndrome. This work-up should include gastroscopy, colonoscopy, gynecological examination, chest CT. A search for malignant cells in the urine may be added to detect urothelioma in patients with frequent use of analgesic drugs.

Although any subtype of primary or secondary GN may be associated with cancer, the risk of solid tumors is more frequent in patients with MN, especially in those older than 60 years of age. As pointed out by several investigators, the diagnosis of MN often precedes that of the associated cancer, sometimes even by years [13, 38]. Therefore, work-up for malignancy in MN should also be initiated in patients with non-nephrotic proteinuria. Some clues may suggest the presence of cancer in a patient with MN, although there are no specific signs or symptoms. When examining the renal biopsy, mesangial or sub-endothelial electron dense deposits are not seen in idiopathic MN and should raise the suspicion of a secondary form of MN. The presence of inflammatory cells is rare in idiopathic MN, while it is frequent in cancer-associated MN. More than 8 leukocytes per glomerulus showed 75 % sensitivity and 92 % specificity for identifying cancer-associated MN [13]. Immunofluorescence microscopy shows that IgG4 deposits are usually seen in idiopathic MN, while deposits of IgG1, IgG2 or IgG3 are more frequent in cases of secondary MN, including those associated with cancer [39]. Circulating anti-phospholipase A2 receptor 1 (anti-PLA2R1) antibodies are frequently seen in idiopathic MN [40], while they are rarely found in cancer-associated MN [41]. Accordingly, an intensive search for cancer should be carried out in MN patients without anti-PLA2R1 antibodies, with prevailing IgG1/IgG2 deposits, or with more than eight inflammatory cells per glomeruli at renal biopsy. Since it is not always possible to exclude the casual association between cancer and glomerular disease, an accurate report of the renal biopsy, including tissue IgG subclasses and number of glomerular inflammatory cells is crucial. However, Qin and colleagues reported that 3 tumor-associated MN patients positive for anti-PLA2R1 antibodies had persistent or relapse of proteinuria despite resection of the tumor [41], suggesting that the presence of anti-PLA2R1 antibodies may indicate their pathogenic role and a casual association between cancer and MN.

Thromboembolic disease is another clinical factor that should raise a suspicion of neoplasia in MN. It is also a well-known complication associated with the nephrotic syndrome, especially when the serum albumin concentration is below 2.8 g/dl [42, 43]. The presence of cancer increases the risk of thromboembolism. About 25 % of patients with cancer-associated MN experienced a thrombotic event [44]. Therefore, if a patient with nephrotic syndrome caused by MN also has deep venous thrombosis or a thromboembolic event, it is plausible, though unproven, that he/she will present a higher probability of having cancer-associated MN. Considering the extraordinary diversity of the neoplasia-MN connection, the search for an underlying neoplasia is limited to screening for the most common types of cancer when no signs or symptoms are present. Among solid tumors, lung, colon, breast, prostate, uterus and stomach cancers are the most frequently observed in patients with MN.

Hematological malignancies, including leukemia and lymphoma, can also occur in patients with MN or other types of glomerular diseases, but they are more frequent in patients with minimal change disease. Full screening for hematological malignancy is warranted in patients with unexplained anemia, monoclonal peak at electrophoresis, hepatomegaly or splenomegaly, enlarged lymph nodes, night sweats, and fever or weight loss. Screening should include a bone marrow biopsy, total body CT scan or positron emission tomography scan.

However, only few centers adopt specific screening for cancer in patients with glomerulopathies. A multi-center analysis of the treatments used in patients with MN was carried out in 15 renal units in Piedmont, Italy [45]. All the centers performed cancer screening, but there were considerable differences in the screening programs. All centers collected a detailed clinical history and carried out physical examination, chest x-ray and complete abdominal ultrasound for each patient. Serological cancer biomarkers were checked in 13 centers, the search for occult blood in the stool was done in 11 centers, while in 9 centers colonoscopy was performed and women underwent breast examination and mammography. Six centers performed gastroscopy, while 5 carried out Papanicolauo (Pap) tests for cervical screening. The most frequently observed tumors were in the kidney, stomach, lung, prostate and colon. This survey from an Italian region in which there is close cooperation among renal units confirms the importance of cancer screening but also highlights the need for clear guidelines to avoid differences in the screening programs [45].

**Fig. 1:**
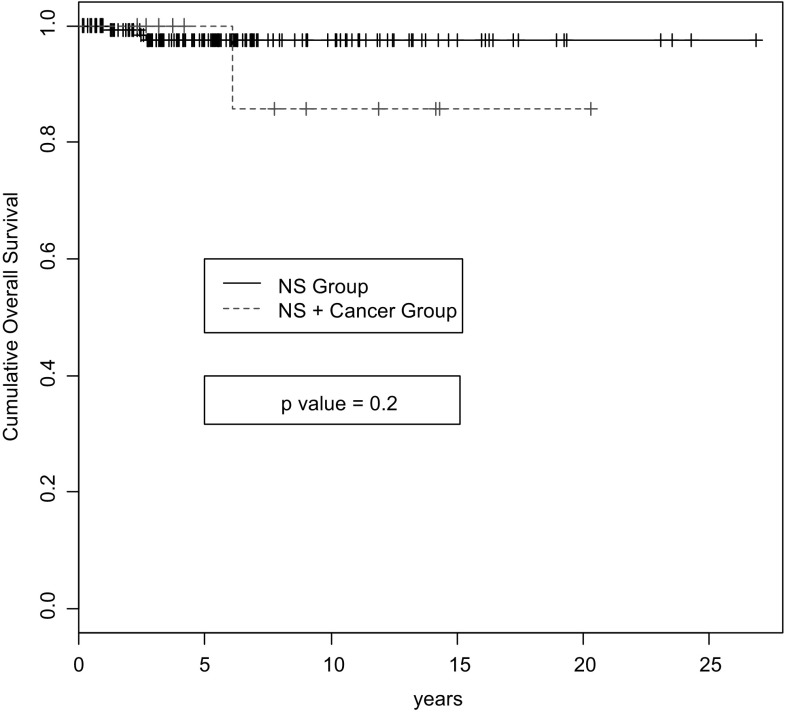
Kaplan-Meier cumulative life survival curves of patients with nephrotic syndrome (NS) and cancer (*red/dashed line*) and patients with nephrotic syndrome alone (*black/continuous line*) in the Renal Unit of the G. Brotzu Hospital in Cagliari, Italy

Few data are available on the screening policies used for GN other than MN. The renal registry of the G. Brotzu Hospital in Cagliari, Italy showed that 163 patients with nephrotic syndrome and biopsy-proven GN underwent “full” screening for cancer from January 1982 to January 2012 (personal communication). The “full” screening performed on all patients included: tumor bio-markers (Ca125, CEA, Ca19.9, Ca15.3, alpha fetoprotein, prostate-specific antigen), stool for occult blood, chest x-ray, complete abdominal ultrasound, gastroscopy, total body CT, colonoscopy, mammography, Pap test for cervical screening and the search for urinary neoplastic cells. Malignancy was discovered in 3 patients (1.8 %). After a median follow-up of 5.7 years (range 1 month–21 years), nine other patients (5.6 %) developed cancer. In total, of the 12 malignancies, seven occurred among 95 patients with MN, and five among 55 patients with MCD. No case of cancer was detected among the six patients with FSGS or the seven with MPGN. The types of cancer are shown in Table 2. We compared the 151 patients without cancer to the 12 patients with cancer by demographic, clinical and renal function data (Table 3). The only significant differences were older age and lower creatinine clearance in patients with cancer. During follow-up, no differences were observed between the two groups with regard to Kaplan–Meier life survival curves (Fig. 1).

**Table 2:** Incidence of neoplasms in 163 patients with nephrotic syndrome undergoing oncological screening at the Renal Unit of the G. Brotzu Hospital in Cagliari, Italy

Cancer	No. patients	Percentage of patients (%)
Breast	2	1.22
Duodenum	2	1.22
Colon-rectum	2	1.22
Basal-cell	1	0.61
Bladder	1	0.61
Kidney	1	0.61
Lung	1	0.61
Thymus	1	0.61
Thyroid	1	0.61
Total	12	7.3

**Table 3:** Baseline characteristics of 163 patients with nephrotic syndrome undergoing oncological screening at the Renal Unit of the G. Brotzu Hospital in Cagliari, Italy

Characteristics	No. patients	Cancer	Control	*p* value
No.	163	12	151	
Male	97	58.3	58.7	0.781
Female	66	41.7	41.3	0.781
Age (years)	54	63	53	**0.049**
Proteinuria (g/24 h)	7	8.2	6.3	0.429
Cholesterol (mg/dl)	304.5	301	327	0.795
Serum albumin (g/dl)	2.4	2.3	2.4	0.570
SCr (mg/dl)	1	1.1	1.0	0.680
Creatinine clearance (ml/min)	96.3	67.8	99.5	**0.046**
eGFR-CKD-Epi (ml/min)	82	71	80.1	0.351
eGFR-MDRD	76	66	74	0.458
Mean arterial pressure (mm/hg)	106	110	104.6	0.305
Follow-up (months)	72	94	72	

The bold values indicate *p* values less than 0.05

*SCr* serum creatinine, *eGFR* estimated glomerular filtration rate, *CKD-Epi* chronic kidney disease epidemiology collaboration formula, *MDRD* modification of diet in renal diseases formula

Since nephrotic syndrome is the clinical renal syndrome that is most often associated with malignancy, we suggest adopting a screening program for nephrotic patients (Table 4). When cancer is detected, the primary treatment must be focused on the cancer in all cases [46].

**Table 4:** Proposed oncological screening of patients with nephrotic syndrome and of patients undergoing long-term immunosuppressive therapy

Screening levels	Proposed procedures
First level analyses	Collection of family and patient’s complete clinical history Careful physical examination including: Skin examination → if suspicious, dermatoscopy Testicular palpation in young males → if positive, testicular US Breast palpation in women → if suspicious, move to a second level test Routine investigations: Complete blood count, PT, PTT, electrolytes, uric acid, and renal and liver function tests, baseline viral titers Chest x-ray Neck US + full abdomen (including renal and urinary tract) US Fecal occult blood search → if positive, gastroscopy ± colonoscopy
Second level analyses (if first level analyses are negative)	Women Gynecological examination → if suspicious, transvaginal US Pap test Breast US ± mammography If unexpected monomorphic hematuria → cystoscopy Men Urological examination (including digital rectal examination) PSA dosage and, if one or both suspicious → trans-rectal prostate US and biopsy If unexpected monomorphic hematuria → cystoscopy
Third level analyses (if first and second level analyses are negative), only in high risk patients (one or more of the following): 1) Heavy smokers 2) Alcohol abusers 3) Older than 60 years/old 4) Thromboembolic events 5) Long term immunosuppressive therapy 6) HBV and/or HCV and/or HIV infection	Colonoscopy Computed tomography of the chest Search for malignant cells in urine and cystoscopy Contrast-enhanced liver US in cirrhotic patients ENT examination ± upper respiratory tract fibroendoscopy Consider cautiously a few specific tumor markers (e.g., alpha_1_-fetoprotein in HBV and/or HCV-positive patients)
Renal pathology clues (only for MN)	High suspicion of secondary MN in case of: Detection of mesangial or sub-endothelial electron dense deposits More than eight leukocytes per glomerulus Prevalence of IgG1, IgG2 or IgG3 deposits at immunofluorescence Absence of anti PLA2R1 antibodies

*US* ultrasound, *PT* prothrombin, *PTT* partial thromboplastin time, *Pap* papanicolauo, *PSA* prostate specific antigen, *HBV* hepatitis B virus, *HCV* hepatitis C virus, *HIV* human immunodeficiency virus, *ENT* ear-nose-throat, *MN* membranous nephropathy, *IG* immunoglobulin, *PLA2R1* phospholipase A2 receptor 1

## For how long should a patient with glomerulopathy be screened for cancer?

Even in the case of a paraneoplastic glomerulopathy, cancer may be clinically discovered years after the diagnosis of renal disease. Indeed, many tumors require years or even decades before exhibiting clinical symptoms. In the meantime, these hidden tumors may release antigens that trigger the production of antibodies leading to the formation of circulating immune complexes or renal deposits of antigens that react with antibodies resulting in local formation of immune complexes. Whatever the mechanism, the deposition of immune complexes may cause inflammation, release of reactive oxygen species and complement activation, possibly leading to glomerular damage.

However, besides the few cases of late recognition of cancer in a paraneoplastic glomerulopathy, one should take into account that glucocorticoids, immunosuppressive agents, or biologic medications that are used to treat chronic GN strongly interfere with the immune response and can favor the development of malignancy or may themselves be carcinogenic [11]. In the absence of guidelines, we recommend that patients who have undergone or undergo long-term immunosuppression should receive complete screening for cancer every 5 years if aged <50–60 years, or every 3 years if they are older.

## Conclusion

Evidence from the literature and from clinical practice suggests that a strong pathogenetic link exists between GN and malignancies. The administration of powerful immunosuppressive drugs, especially cytotoxic drugs and calcineurin inhibitors, may strongly impair the immune response and increase the oncogenic risk. Setting up appropriate screening programs is thus recommended, especially in elderly patients, in those with nephrotic syndrome and in those who undergo long-term immunosuppressive therapy. In the absence of internationally approved guidelines and evidence-based indications, we propose a screening program based on expert opinion, in an effort to help the nephrologist in the early detection of malignancies during clinical practice.
